# Investigation of Different Emulsion Systems on the Performance of Microcapsules Based on Numerical Simulation

**DOI:** 10.3390/ma19071385

**Published:** 2026-03-31

**Authors:** Zihou Tian, Mingxian Liu, Yukang Zheng, Pengfei Ban, Jiace Xue, Jinliang An

**Affiliations:** 1 School of Civil Engineering, Hebei University of Engineering, Handan 056038, China; 13931999594@163.com (Z.T.); 15733929296@163.com (Y.Z.); 15097353327@163.com (P.B.); 13472016148@163.com (J.X.); 2 School of Mechanical and Equipment Engineering, Hebei University of Engineering, Handan 056038, China; 18712909003@163.com

**Keywords:** microencapsulation, agitation process, capsule particle size, CFD simulation, cavitation phenomenon

## Abstract

During microencapsulation, agitation is typically required to achieve the homogeneous dispersion of the reaction mixture, with the mixing and dispersion efficiency within the reactor being predominantly determined by the rotational speed. However, when the agitation speed exceeds a certain threshold, cavitation occurs during the stirring process. This cavitation phenomenon can significantly influence the properties of the resulting microcapsules. Therefore, this study combines the CFD simulation method with microcapsule preparation experiments, focusing on the occurrence of cavitation during the stirring process and its effect on the particle size of the prepared microcapsules. The CFD simulations analyzed flow field characteristics under different agitation speeds within the beaker, including phase distribution contours, streamline patterns, turbulent kinetic energy fields, and shear stress distributions. Different fluid flows were established by changing the rotating speed of the paddle, and the influence of each fluid flow on the particle size and distribution of the prepared microcapsules was determined. Particular emphasis was placed on examining the influence of rotational speeds ranging from 550 to 850 rpm on microcapsule particle size. Experimental validation confirmed that the impeller speed of 650 rpm provided superior flow field control, yielding microcapsules with the narrowest particle size distribution. This study elucidates the mechanism through which cavitation influences the microencapsulation process, thereby providing both theoretical insights and experimental support for the optimization of microcapsule preparation techniques.

## 1. Introduction

In recent years, microencapsulation technology has been widely employed for the immobilization of active substances and chemical pharmaceuticals, finding extensive applications across diverse fields including industrial processing, textiles, pharmaceuticals, food processing, and electronic information [1,2,3,4,5]. This technology demonstrates significant application potential and market value. By encapsulating active ingredients within minute yet stable shell structures, microencapsulation not only effectively protects core materials from environmental influences but also enables controlled release characteristics through tailored shell properties. This allows for the precisely timed, quantified, and targeted release of active components to meet complex application requirements [6,7,8]. Particle size, as one of the fundamental characteristics of microcapsules, significantly influences their performance and application efficacy. The dimensions of microcapsules directly determine critical parameters including specific surface area, permeability, structural stability, and release kinetics, which subsequently govern their dispersibility, targeting capability, biocompatibility, and ultimate functional performance in specific environments [9,10,11,12]. Consequently, precise control of particle size represents a crucial technical challenge in microcapsule fabrication processes. Kosarli et al. [13] regulated the agitation speed to investigate the mechanical properties, thermal stability, and self-healing efficiency of microcapsules with five distinct mean diameters. Their findings revealed that smaller microcapsules maintained thermal stability at elevated temperatures up to approximately 230 °C. Xue et al. [14] employed microfluidic technology to fabricate highly uniform microcapsules using boron nanoparticles as the core material and chitosan as the shell matrix. The resulting microcapsules effectively prevented the agglomeration of boron nanoparticles, shortened mass transfer distances, and enhanced the contact area between boron nanoparticles and metal catalysts, thereby significantly improving combustion efficiency. Luo et al. [15] demonstrated that the dispersal and release behavior of pesticide microcapsules in field applications can be modulated simply by adjusting their particle size, a characteristic particularly valuable for agricultural applications. Dong et al. [16] systematically analyzed the properties influenced by microcapsule size, showing that increased microcapsule diameter led to greater shell thickness and enhanced swelling capacity. These studies collectively confirm that microcapsule size exerts a profound influence on release kinetics and overall performance, underscoring the necessity of precise particle size control in microcapsule design and application.

Current encapsulation technologies for phase change materials (PCMs) encompass various approaches, including in situ polymerization [17], interfacial polycondensation [18], as well as suspension and emulsion polymerization [19]. While these methods generally provide PCMs with reliable chemical stability, they present certain limitations such as inadequate mechanical strength, prolonged experimental durations, and the inability to directly observe internal system dynamics. In recent years, advancements in computational technology have driven significant progress in computational fluid dynamics (CFD), which has become widely adopted for simulating complex fluid flow phenomena [20,21]. Compared to conventional experimental methods, numerical simulations not only enable the rapid and convenient acquisition of reliable results with substantially reduced experimental cycles, but also demonstrate remarkable advantages in conserving manpower, material resources, and time investments. Over recent decades, computational fluid dynamics (CFD) has been increasingly utilized to investigate fluid flow behavior in stirred tank reactors [22,23]. Zamiri et al. [24] employed the Scale-Adaptive Simulation (SAS) approach via the commercial computational fluid dynamics (CFD) software ANSYS Fluent, to numerically analyze turbulent structures within a stirred tank equipped with a Rushton turbine. Their computational results demonstrated that the SAS method effectively predicted both radial and tangential velocity components, while providing reasonable estimations of Reynolds stresses and turbulent kinetic energy. In a separate study, Mittal et al. [25] conducted comparative analysis between experimental measurements and simulation data, revealing that CFD simulations successfully captured the overall flow characteristics while exhibiting certain discrepancies in specific flow features. These findings underscore the importance of selecting appropriate turbulence models in CFD simulations to ensure computational accuracy and reliability. Torré et al. [26,27] conducted experimental and numerical investigations on the transient shutdown process of a gas–liquid stirred tank equipped with baffles. Their results demonstrated good agreement between experimental data and simulations. At higher impeller speeds, the vortex generated by the impeller entrained upper gas-phase media into the lower liquid-phase region, with the affected zone of the gas phase expanding as rotational speed increased. Wadnerkar and Guan et al. [28,29] systematically evaluated four different drag models for simulating gas–liquid two-phase flow in stirred tanks. Their research emphasized the critical importance of drag model selection for accurate prediction of gas–liquid hydrodynamics, while also identifying the impeller zone as the region of maximum turbulent kinetic energy. Zhao et al. [30] examined the effects of impeller geometry, reactor scale, and agitation speed on the resultant microcapsule size. Through CFD simulations of flow characteristics under varying structural and operational parameters, coupled with correlation analysis between simulation results and experimental data, they established a negative linear relationship between average flow velocity and mean microcapsule diameter. Their findings indicate that microcapsule size can be effectively controlled by adjusting agitation intensity, with higher stirring speeds producing smaller microcapsules. However, excessively high agitation speeds can induce unstable flow patterns within the reactor due to the intense rotational motion of the impeller. Yoon et al. [31] demonstrated that extreme agitation intensities generate cavitation phenomena in stirred tanks, leading to material accumulation around the impeller. To address this challenge, they proposed the innovative integration of mesh structures within the agitation system to stabilize fluid motion, thereby enabling the production of microcapsules with superior size uniformity.

In summary, while most of the reported studies mainly adopt steady-state CFD simulations to investigate the flow field distributions during microencapsulation preparation, the influence of flow field variations during agitation on the homogeneity and performance analysis of the as-prepared microcapsules remains unclear, and the application of transient simulation methods is rarely reported in this field. To address these research gaps, this study adopts a technical route that combines CFD transient numerical simulation with in situ polymerization microcapsule preparation experiments, aiming to reveal the mechanism through which flow field distribution affects the particle size of the prepared microcapsules under the condition without active agitation speed control.

## 2. Experimental Materials and Preparation Methods

### 2.1. Raw Materials

All the raw materials, specifications, manufacturers and core uses of epoxy resin microcapsules prepared in this study are shown in Table 1.

### 2.2. Microencapsulation Process

Epoxy resin microcapsules were prepared via in situ polymerization, with the core procedure split into three sequential stages: MUF prepolymer synthesis, core emulsion preparation, and in situ polymerization curing. Detailed steps are as follows:

1. Synthesis of MUF Prepolymer

Into a three-necked beaker with 700 g deionized water, 60 g U, 180 g F and 2 g solid sodium hydroxide were added sequentially. The mixture was stirred and heated to 50 °C for full dissolution, then 15 g M was introduced and heated to 70–75 °C. Sodium hydroxide solution maintained a weak alkaline environment, and constant stirring continued until the solution clarified to form MUF prepolymer, which was cooled and sealed for standby.

2. Preparation of Core Material Emulsion

A fixed amount of deionized water was added to an emulsification reactor, followed by 2.4 g sodium chloride, 6.75 g resorcinol, 27 g PVA and 135 g EMA polyethylene maleic anhydride. The mixture was stirred and heated to 46 °C until dissolved, then quantitative epoxy resin core material was added. Shear dispersion at a set speed for a fixed time generated a stable oil-in-water epoxy emulsion.

3. In Situ Polymerization and Fabrication of Epoxy Resin Microcapsules

The prepared emulsion was transferred to a temperature-controlled reactor at 41 °C. Under constant stirring, MUF prepolymer was slowly dripped, with dilute sulfuric acid adjusting pH. After adding prepolymer, the mixture reacted isothermally for 30 min. pH was fine-tuned with sodium hydroxide, while 30 g KH570 was pre-hydrolyzed in 60 g deionized water for 30 min and then dripped in. The reaction persisted for another 30 min, followed by heating and constant-temperature reaction for 3 h to finish curing.

**Figure 1:**
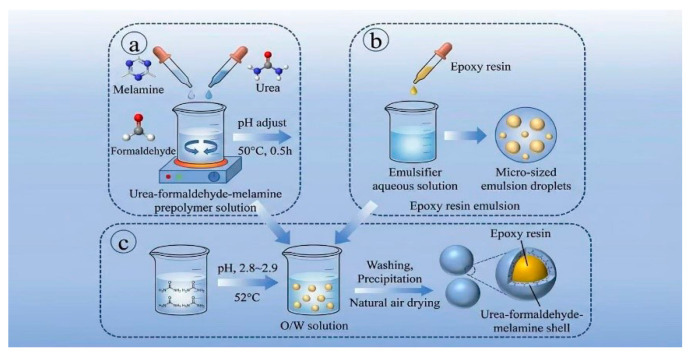
Schematic diagram of the microencapsulation process (schematic illustration of the ex-perimental preparation procedure). (**a**) Synthesis of MUF Prepolymer; (**b**) Preparation of Core Material Emulsion; (**c**) In Situ Fabrication of Epoxy Resin Microcapsules.

After reaction, the product was washed with deionized water and anhydrous ethanol, vacuum-filtered, and air-dried at room temperature to obtain final microcapsules. The core preparation mechanism and procedure are displayed in Figure 1.

### 2.3. Characterization Technique

This study systematically characterized the microcapsules in terms of particle size distribution, micromorphology, core material encapsulation efficiency and thermal stability. The instruments, sample preparation methods and test conditions are listed as follows:

1. Particle Size Distribution Test

The particle size distribution of microcapsules was measured using a Mastersizer 3000 laser particle size analyzer (Malvern Instruments Ltd., Malvern, UK).

2. Micromorphology Characterization

The surface morphology, sphericity and damage of microcapsules were observed via a binocular optical microscope (XSP-2CA, Shanghai Youke Instrument Co., Ltd., Shanghai, China).

## 3. Numerical Simulation

### 3.1. Simulation Physical Setup and Dimensionless Numbers

The simulation system is defined as a gas–liquid two-phase flow, with physical parameters of both phases adopting measured values under standard atmospheric pressure. The gas–liquid interfacial tension coefficient is set to 0.07 N/m, the pressure reference is standard atmospheric pressure, and gravitational acceleration is assigned as $g=-9.81\;m/s^{2}$ along the negative *Y*-axis direction. A transient simulation scheme is adopted, with the stirring speed ranging from 550 to 850 rpm.

To quantitatively characterize the flow behavior of the stirring system, two dimensionless numbers, namely the stirring Reynolds number (Re) and Weber number (We), are introduced in this study. The calculation formula of the stirring Reynolds number is expressed as follows:

$$Re=\frac{\rho nd^{2}}{\mu }$$

where ρ is the liquid phase density $\left(kg/m^{3}\right)$, *n* is the stirring impeller speed $\left(r/s\right)$, *d* is the impeller diameter, and μ is the liquid dynamic viscosity $\left(Pa\cdot s\right)$.

The calculation formula of the Weber number is given as follows:

$$We=\frac{\rho n^{2}d^{3}}{\sigma }$$

where σ is the surface tension coefficient of gas–liquid two-phase $\left(N/m\right)$.

As listed in Table 2, the Reynolds numbers under all working conditions in this study exceed $10^{4}$, indicating a fully developed high-Reynolds turbulent flow throughout the process. Among two-equation turbulence models, the Standard k-ε model features the optimal computational stability and fastest convergence rate. Compared with high-order models, it significantly reduces computational cost while ensuring accuracy, avoiding convergence failures in multi-case transient simulations.

### 3.2. Numerical Simulation Method

This study performs three-dimensional full-scale CFD transient simulations of gas–liquid two-phase flow in a stirred tank. The Volume of Fluid (VOF) model is adopted, with the Standard k-ε turbulence model for fully turbulent flow, Dynamic Mesh (DM) for impeller rotation, and Finite Volume Method (FVM) for solving governing equations.

A complete mathematical governing system is built for transient gas–liquid flow, revealing the hydrodynamic effects of rotating walls; detailed equations and analysis are presented in Section 3.7.

### 3.3. Geometric Modeling

**Figure 2:**
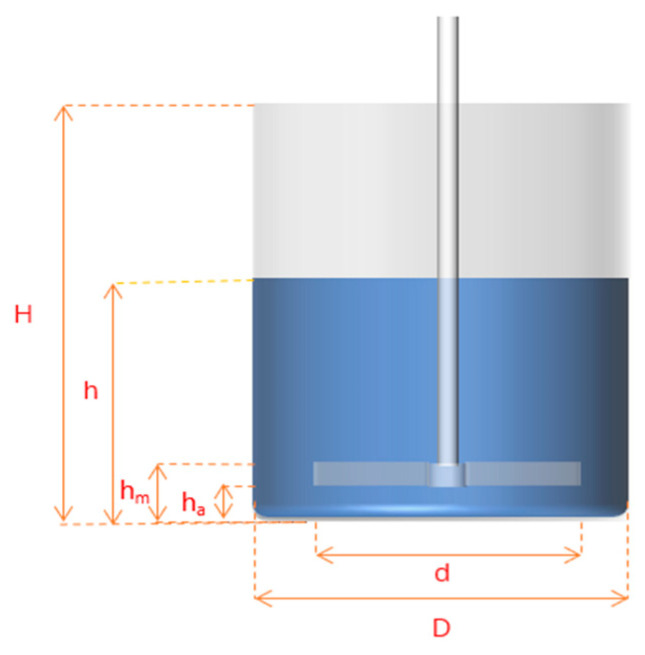
Schematic diagram of the emulsification and agitation equipment for microcapsule preparation (geometric model for numerical simulation).

Figure 2 shows the geometry of the stirred tank and impeller. Key dimensions are defined as: Tank inner diameter (D), impeller diameter (d), total tank height (H), solution filling height (h), blade height (h_m_), and impeller-bottom distance (h_a_). Detailed values are listed in Table 3.

The impeller is placed 5 mm above the tank bottom, with solution filled to 50 mm height. The three-bladed impeller (120° spacing) has a 40 mm diameter, with blade height, length and thickness of 5 mm, 20 mm and 1 mm, respectively.

**Figure 3:**
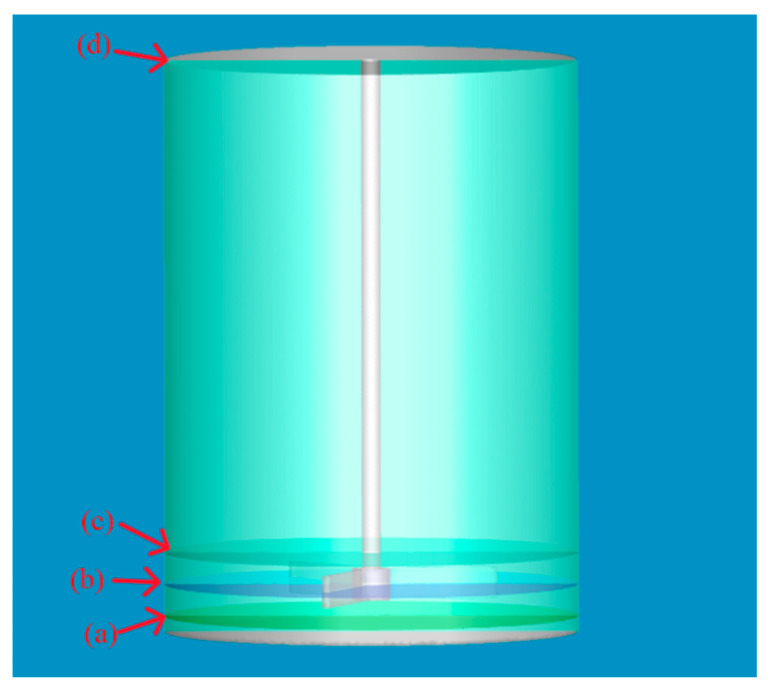
Reference planes for flow field analysis in the emulsification and agitation system (numerical simulation model): (a) Horizontal plane 2.5 mm above the reactor base; (b) horizontal plane 7.5 mm above the reactor base; (c) horizontal plane 12.5 mm above the reactor base; (d) 0° vertical plane through the agitator centerline.

Figure 3 presents the analysis reference planes: The 0° plane runs through the blade tip and center. Data are extracted from planes at +2.5 mm, +7.5 mm and +12.5 mm along the *z*-axis (tank bottom centerline: z = 0).

### 3.4. Mesh Generation

The computational domain is split into two regions: The impeller rotation zone (momentum source loading area) and the static fluid zone. The cylindrical rotation zone is driven by the impeller’s rigid-body rotation (via dynamic mesh) and momentum sources synchronously.

Unstructured tetrahedral meshes are used for domain discretization to fit the complex impeller geometry and satisfy mesh deformation requirements of the dynamic mesh model, ensuring simulation reliability.

Four gradually refined mesh schemes are designed for grid independence verification (Table 4). A second-order volume-weighted interpolation (cell centroid-based) is adopted for cross-mesh flow field comparison; second-order upwind face interpolation and inverse distance-weighted node interpolation are reserved for non-coincident nodes and local dynamic mesh deformation, respectively.

**Figure 4:**
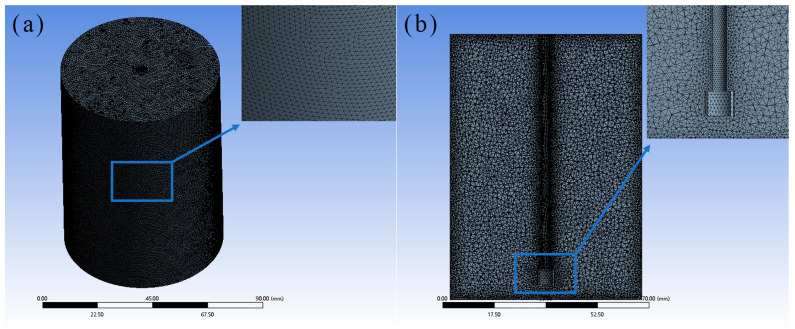
Computational grid of the emulsification and agitation equipment for numerical simulation: (**a**) Front view of the full computational domain; (**b**) cross-sectional view of the grid.

As listed in Table 4, all four mesh schemes exhibit excellent quality: The minimum orthogonality quality is ≥0.28 and the maximum aspect ratio is ≤5.3, fully satisfying the accuracy requirements for transient turbulent and gas–liquid two-phase flow simulations in the stirred tank. Local mesh refinement is performed at the blade tip and gas–liquid interface to capture subtle flow variations, and the grid layout and cross-sectional distribution are displayed in Figure 4.

Grid convergence tests were conducted following CFD standards to eliminate mesh size effects and verify grid independence, with results listed in Table 5.

Table 5 shows that under the benchmark condition of 650 r/min, as the element size is refined from 1.2 mm to 0.8 mm, the variation rates of the three core flow field parameters gradually decrease and converge. When the mesh is refined to 0.6 mm, the maximum Grid Convergence Index (GCI) is only 0.42% (<5%), indicating negligible discretization error and strict grid independence. The GCI of the 0.8 mm mesh is 1.08%, which also meets the convergence criterion and significantly reduces computational cost compared with the 0.6 mm mesh, achieving a balance between accuracy and efficiency.

Table 6 indicates that at the maximum rotational speed of 850 r/min, the maximum GCI is only 0.38% (<5%) for the 0.6 mm mesh, and 0.96% for the 0.8 mm mesh, both satisfying the 5% convergence threshold. The relative deviation of maximum turbulent kinetic energy between 0.8 mm and 0.6 mm meshes is only 1.08%, confirming the strict grid independence of turbulent kinetic energy calculation at the maximum speed. Thus, the 0.8 mm element size mesh is adopted for all working conditions to ensure the reliability of cross-condition comparison of turbulent kinetic energy and other flow field parameters within 550~850 r/min.

### 3.5. Boundary Conditions and Source Term Quantification for Impeller Motion

**Figure 5:**
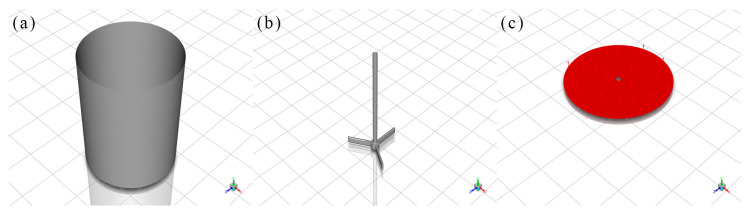
Schematic of computational domain boundaries for the emulsification stirring device (numerical simulation setup): (**a**) Stirred tank wall; (**b**) impeller blade wall and hub wall; (**c**) top gas outlet of the computational domain.

The computational domain of stirred tank is divided into three categories: solid boundary (bottom, side wall of stirred tank, impeller wall), gas boundary (gas-liquid free surface, gas outlet at the top of computational domain) and internal interface (dynamic grid interface between impeller rotating area and fluid domain), in which the impeller wall adopts non-slip wall boundary, and the driving effect of impeller rotation on fluid is quantified and realized through the momentum source term of rotating area. The schematic diagram of calculation domain boundaries is shown in Figure 5, and the positions, types and setting parameters of each boundary are shown in Table 7.

In this study, impeller rotation is numerically quantified by the volume force source term in the momentum equation. The impeller speed is converted into a momentum source term within the rotating zone to drive the matching flow, and the transient two-way coupling between rigid impeller rotation and flow field evolution is realized combined with the dynamic mesh model (Figure 5) (Table 7).

The actual impeller speed *n* is converted into the rotational angular velocity vector $\vec{\omega }$, with the quantification formula:

$$\vec{\omega }=\frac{2\pi n}{60}\cdot \vec{e_{y}}$$

In the absolute reference frame, the force induced by rotational motion is characterized by the volume force source term ${\vec{F}}_{rotation}$ in the momentum equation, consisting of centrifugal and Coriolis force source terms:

$${\vec{F}}_{rotation}={\vec{F}}_{centrifugal}+{\vec{F}}_{coriolis}$$

### 3.6. Solution Method and Time Step Independence Verification

In this study, the Finite Volume Method (FVM) is used for spatial discretization of all governing equations. Featuring strict physical conservation via control volume integration, FVM is a standard method for rotating machinery turbulent flow simulation [32,33]. All numerical calculations, dynamic mesh operations and post-processing are performed on ANSYS Fluent 2022 R1, whose mature dynamic mesh module well satisfies the transient stirred tank flow simulation requirements.

FVM boasts clear physical significance, low computational cost, favorable convergence and simple boundary treatment. The gas–liquid two-phase flow equations with rotational momentum source terms (continuity, momentum, k−ε turbulence and VOF equations) are discretized on unstructured tetrahedral meshes, with a second-order upwind scheme for convective terms, a central difference scheme for diffusive terms, and PISO algorithm for transient pressure-velocity coupling.

A single time step converges when all equation residuals drop below 10^−4^, and key parameters (average fluid velocity, turbulent kinetic energy, liquid level height) stabilize over time.

For transient unsteady flow simulation, the time step follows the impeller rotation angle criterion: The single-step rotation angle is limited to ≤1° to ensure flow field accuracy, a common standard for stirred tank simulations that balances precision and efficiency.

According to the impeller angular velocity and rotation angle criterion, the calculation formula of the time step Δt is as follows:

$$∆t\leq \frac{∆\theta }{360\cdot n/60}=\frac{∆\theta }{6n}$$

where Δθ=1° is the maximum rotation angle per step, and *n* is the stirring speed (rpm).

Calculated maximum allowable time steps for each speed: 550 rpm (≤3.03 × 10^−4^ s), 650 rpm (≤2.56 × 10^−4^ s), 750 rpm (≤2.22 × 10^−4^ s), 850 rpm (≤1.96 × 10^−4^ s). A fixed time step of $\Delta t=2.0\times 10^{-4}s\;$ is adopted for all cases, satisfying the ≤1° rotation angle requirement.

To eliminate time discretization errors and verify time step independence, three schemes are tested at 650 rpm, with average velocity, maximum blade-tip TKE and stable liquid level as evaluation indicators (Table 8).

As shown in Table 8, reducing the time step from 2.0 × 10^−4^ s to 1.0 × 10^−4^ s yields a mere 0.68% average relative deviation of key parameters, far below the 5% engineering tolerance, proving strict time step independence. The 2.0 × 10^−4^ s step delivers consistent results with lower computational cost, balancing accuracy and efficiency, and is thus selected for all simulations.

### 3.7. Governing Equations

#### 3.7.1. General Governing Equations

This study uses the VOF model to capture gas–liquid interfacial behavior and the Standard k-ε model for turbulence characterization. A complete mathematical model for gas–liquid flow in the stirred tank is constructed via transient continuity and momentum equations, with the hydrodynamic effect of the rotating impeller wall clarified as follows.

Transient continuity equation:

$$\frac{\partial \rho }{\partial t}+\nabla \cdot \left(\rho \vec{u}\right)=0$$

where ρ is the fluid density $\left(kg/m^{3}\right)$, t is the time (s), and $\vec{u}$ is the fluid velocity vector (m/s).

Transient momentum equation:

$$\frac{\partial \left(\rho \vec{u}\right)}{\partial t}+\nabla \cdot \left(\rho \vec{u}\vec{u}\right)=-\nabla p+\nabla \cdot \left(\mu_{eff}(\nabla \vec{u}+\nabla {\vec{u}}^{T})\right)+\rho \vec{g}+{\vec{F}}_{total}$$

where p is the hydrostatic pressure (Pa), $\mu_{eff}$ is the effective dynamic viscosity (Pa·s), $\vec{g}$ is the gravitational acceleration vector $\left(m/s^{2}\right)$, and ${\vec{F}}_{total}$ is the total volume force vector $\left(N/m^{3}\right)$. In this study, the surface tension between gas and liquid phases is mainly considered.

#### 3.7.2. VOF Multiphase Flow Governing Equations

The VOF model tracks phase volume fractions to depict gas–liquid distribution and interface features. The transport equation for the q phase volume fraction is:

$$\frac{\partial \alpha_{q}}{\partial t}+\vec{\mu }\cdot \nabla \alpha_{q}=0$$

where $a_{q}$ is the volume fraction of the q phase fluid, satisfying $\sum_{q=1}^{n}\alpha_{q}=1$ (n is the number of phases, n=2 in this study, representing the aqueous phase and gas phase respectively).

#### 3.7.3. Standard k-ε Turbulence Governing Equations

The Standard k-ε model solves transport equations for turbulent kinetic energy k and dissipation rate ε to describe turbulence. Effective viscosity: $\mu_{eff}=\mu +\mu_{t}$, with turbulent viscosity $\mu_{t}=\rho C_{\mu }\frac{k^{2}}{\varepsilon }$ ($C_{\mu }$ = 0.09, empirical constant).

## 4. Results and Discussion

Note on the consistency unification of flow field snapshots: In this study, all instantaneous snapshots of flow field parameters and quantitative radial velocity data under different rotational speeds are extracted at the identical physical time t=20 s This ensures the consistency of the flow field development duration and the rigor of cross-condition comparison.

Due to the inherent difference in the angular velocity of the stirring paddle under different rotational speeds, the rotation angle of the paddle within the same physical time is different, resulting in the difference in the circumferential position of the paddle section in each subfigure. This difference in paddle position is solely caused by the physical characteristics of the rotational speed itself and does not affect the lateral comparison of the macroscopic characteristics, statistical rules and core parameters of the flow field under different working conditions.

**Figure 6:**
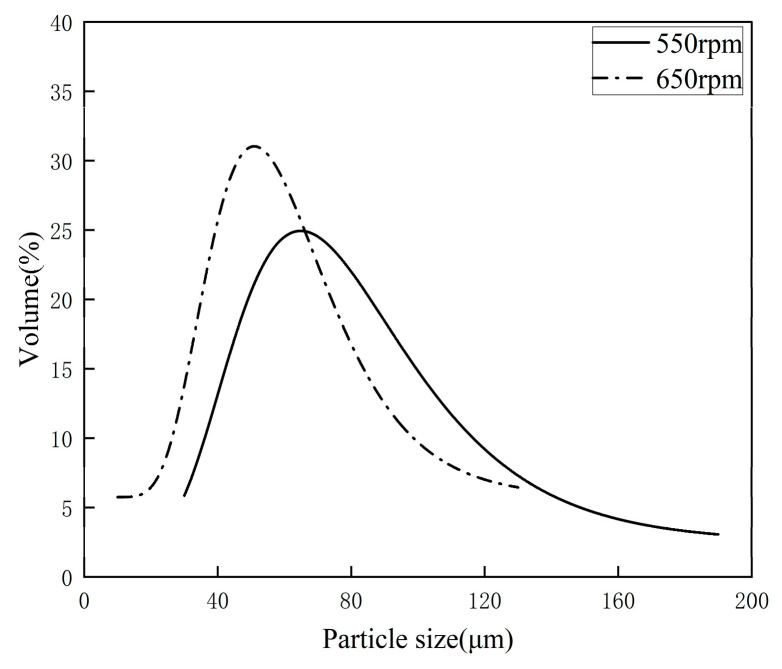
Particle size distribution of microcapsules prepared at different agitation speeds (experimental results).

Figure 6 presents the particle size distribution of microcapsules prepared at various stirring speeds. The stirring speed serves as a core process parameter that governs the dispersion of emulsion droplets, as well as the final particle size and distribution uniformity of microcapsules.

Differences in shear action and flow field conditions under varying speeds directly lead to remarkable discrepancies in the particle size and distribution width of microcapsules.

Experimental results reveal that the average emulsified particle size of microcapsules is 130.33 μm at 550 rpm. When the speed increases to 650 rpm, the average particle size decreases to 120.74 μm, accompanied by a higher particle size distribution peak, indicating a more uniform size distribution of the as-prepared microcapsules at 650 rpm.

**Figure 7:**
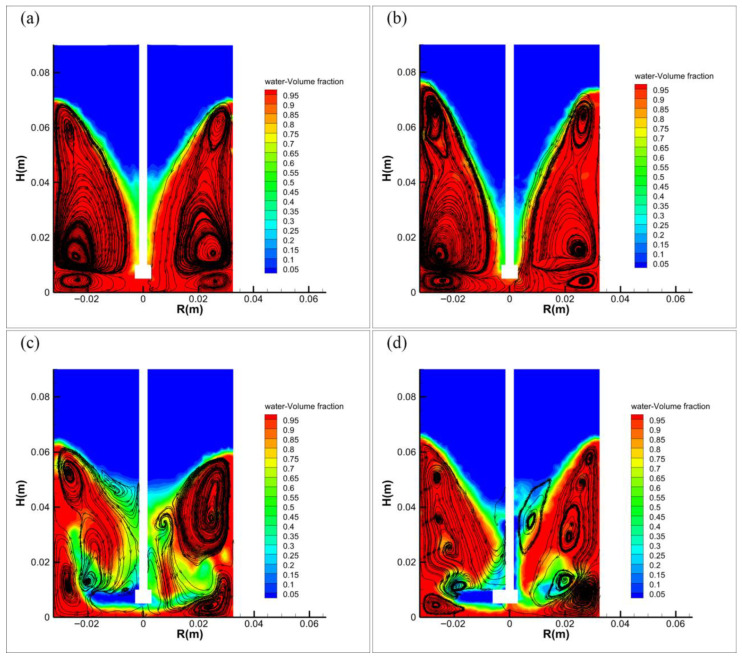
Gas–liquid two-phase volume fraction contours and streamline distributions at different agitation speeds (instantaneous snapshots from numerical simulation, captured at t = 20 s): (**a**) 550 rpm, (**b**) 650 rpm, (**c**) 750 rpm, (**d**) 850 rpm.

**Figure 8:**
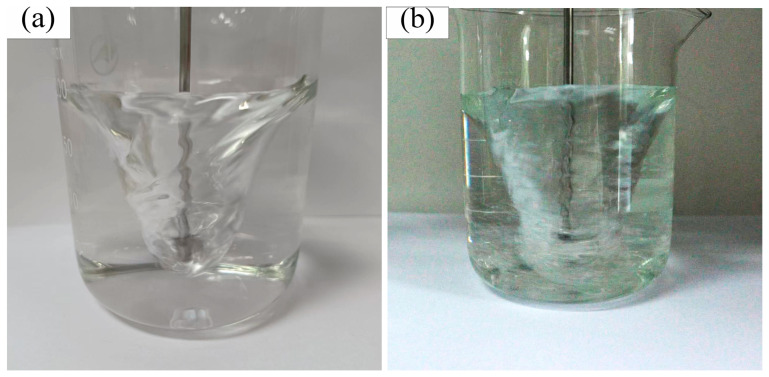
Bubble distribution patterns at different rotational speeds (instantaneous snapshots from experiments): (**a**) 650 rpm, (**b**) 750 rpm.

Figure 7 shows the gas–liquid two-phase volume distribution contours and streamlines at different rotational speeds, where red (lower part) represents water, blue (upper part) represents air, and black arrows represent liquid flow trajectories (streamline plot). Figure 7 demonstrates that at 550 rpm, the funnel-shaped bottom of the gas–liquid interface begins to reach the center of the impeller. When the rotational speed increases to 650 rpm, the air phase extends downward and reaches the bottom of the impeller center. At this stage, the number of large-diameter vortices in the system remains stable, and two distinct vortices form beneath the impeller, creating a self-circulation zone that contributes to relative stability in the agitation system. As the rotational speed further increases to 750 rpm, the air phase begins to reach the vicinity of the impeller tips due to the intensified rotation. The intrusion of air causes the large-diameter vortices to break down into smaller ones, and the self-circulation zone beneath the impeller starts to shift outward, resulting in flow field destabilization. Figure 8 provides a more direct visualization of how gas penetration into the flow field causes flow destabilization, as continued increase in rotational speed keeps the air phase near the impeller region and progressively multiplies the number of vortices, leading to further flow field disorganization.

During the agitation process, fluid energized by the impeller propagates radially toward the vessel walls. Upon wall impingement, the flow diverges into upward and downward streams, generating characteristic vortex structures. The clustered streamlines beneath the gas–liquid interface conform to the interface morphology, forming stable vortices that subsequently induce secondary liquid circulation. This secondary flow phenomenon can be attributed to the uneven gas holdup distribution resulting from excessive impeller rotational velocity, as documented in reference [25]. As shown in the results, the number of vortices remains essentially constant and symmetrically distributed at rotational speeds below 750 rpm, where the orderly rotation of the liquid phase within these vortices enables more convenient control of droplet size. However, when the speed exceeds 750 rpm, air penetration into the reaction system causes the breakdown of large-diameter vortices and subsequent flow field destabilization; the intensified collision between the liquid phase and impeller leads to excessive droplet fragmentation, which adversely affects the control of microcapsule size during the reaction process.

The critical rotational speed for vortex breakdown holds significant implications for microencapsulation systems. Therefore, to achieve rational and convenient control of microcapsule particle size during preparation, it is essential to prevent vortex disintegration by maintaining the impeller speed below 750 rpm.

**Figure 9:**
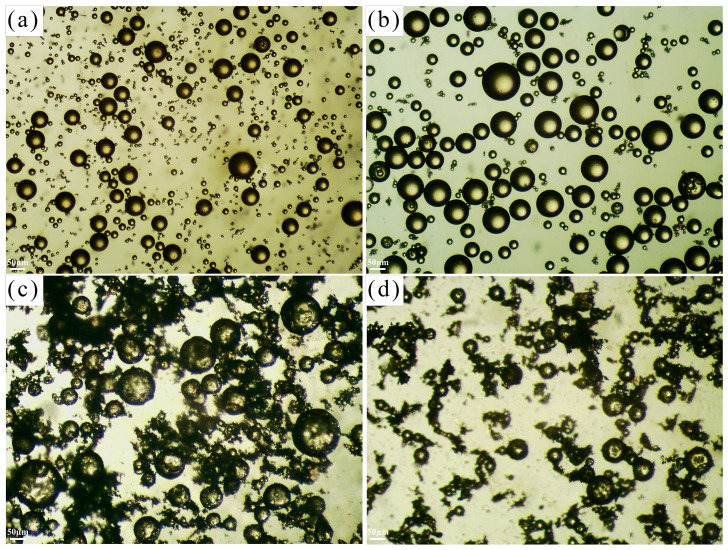
Optical microscope images of microcapsules prepared at different agitation speeds (experimental results): (**a**) 550 rpm, (**b**) 650 rpm, (**c**) 750 rpm, (**d**) 850 rpm.

The influence of agitation speed on the particle size and distribution of the prepared microcapsules was investigated within the range of 550 to 850 rpm (Figure 9). The results indicate that both the particle size and distribution uniformity of the microcapsules decline with increasing agitation speed, reaching the optimal value at 650 rpm. As the rotational speed rises, bubbles form in the reaction liquid, creating low-pressure regions within the bubbles. These bubbles rapidly collapse under the action of the surrounding high-pressure liquid phase, causing flow field disturbances that enhance turbulence and shear forces. This intensified hydrodynamic environment leads to the fragmentation of larger microcapsules, resulting in reduced overall particle size and broader size distribution. As observed in Figure 9, microcapsules prepared at 650 rpm exhibit superior size uniformity. When the rotational speed increases to 750 rpm, the microcapsules begin to show irregular particle sizes and difficulties in maintaining structural integrity. Further escalation to 850 rpm results in widespread size heterogeneity and significant challenges in microcapsule formation. Therefore, to obtain microcapsules with consistent particle size distribution, an optimal agitation speed of 650 rpm should be employed.

**Figure 10:**
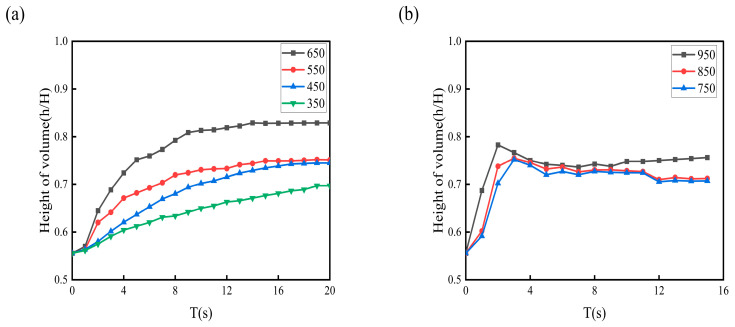
Temporal variations in fluid height within the agitated vessel (numerical simulation results): (**a**) rotational speeds of 350–650 rpm; (**b**) rotational speeds of 750–950 rpm.

Figure 10 reveals that below 650 rpm, the liquid level height gradually increases with rotational speed and stabilizes after a certain period. When the speed exceeds 750 rpm, the liquid level experiences a sudden drop at a specific moment. This phenomenon occurs because bubbles initially form at the tip of the impeller’s suction side and progressively expand across the entire suction surface as rotational speed increases. The gas holdup near the impeller and beneath it continues to intensify. While the fundamental funnel-shaped geometry remains largely unchanged, the increasing gas accumulation beneath the impeller causes the funnel structure to gradually shift upward, consequently leading to the observed liquid level depression.

Combined with Torré’s [26,27] research on time-dependent gas–liquid distribution in stirrers, this study further clarifies the transient bubble formation mechanism: Higher rotation speed instantly strengthens impeller shear, non-uniformly entraining top gas to the impeller zone, while transient bubble nucleation and collapse disturb flow field stability and impeller parameters.

Air ingestion triggers transient flow disturbance during stirring, which breaks fluid local equilibrium, damages droplet surface tension and intermolecular forces, consumes stirring mechanical energy, increases droplet friction and ultimately weakens stirring efficiency.

The intensity and frequency of transient disturbance directly determines system stability and microcapsule particle size distribution. The 650 rpm condition features the lowest disturbance intensity and most stable frequency, thus yielding a narrower particle size distribution.

**Figure 11:**
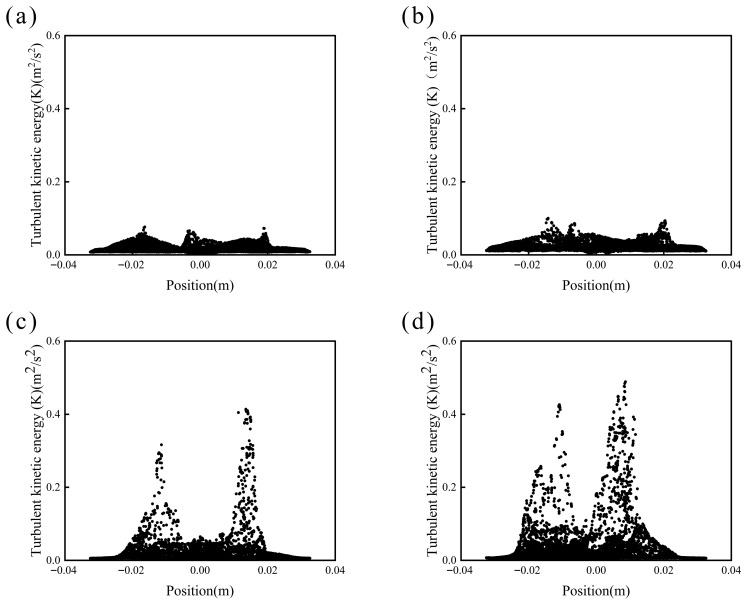
Turbulent kinetic energy (TKE) distribution on the horizontal plane 7.5 mm above the reactor base at different rotational speeds (numerical simulation, captured at t = 20 s): (**a**) 550 rpm, (**b**) 650 rpm, (**c**) 750 rpm, (**d**) 850 rpm.

Furthermore, the turbulent kinetic energy (TKE) at the 7.5 mm cross-section (Figure 11) was analyzed. The results reveal significant differences within the reactor. At speeds up to 650 rpm, TKE values are primarily concentrated in the range of 0.01–0.03 m^2^/s^2^. In contrast, at 750 rpm and higher speeds, TKE values mainly distribute between 0 and 0.1 m^2^/s^2^. Moreover, as the rotational speed increases, the maximum turbulent intensity rises sharply, with peak values predominantly localized near the impeller tip region. Under non-cavitating conditions, the turbulent kinetic energy (TKE) distribution remains uniform, with recorded maximum TKE values of only 0.0765 m^2^/s^2^ and 0.1001 m^2^/s^2^. In general, TKE is directly correlated with fluid dispersion and consequently influences the particle size distribution of the prepared microcapsules. The more homogeneous TKE distribution in the absence of cavitation contributes to a narrower particle size distribution, which aligns well with the experimental observations. Comparative analysis with experimental results confirms that under cavitation-free conditions, the system at 650 rpm demonstrates more uniform emulsion dispersion compared to 550 rpm. This enhanced homogeneity promotes better distribution and interaction between the core and shell materials, thereby facilitating improved microencapsulation efficiency and coating integrity.

**Figure 12:**
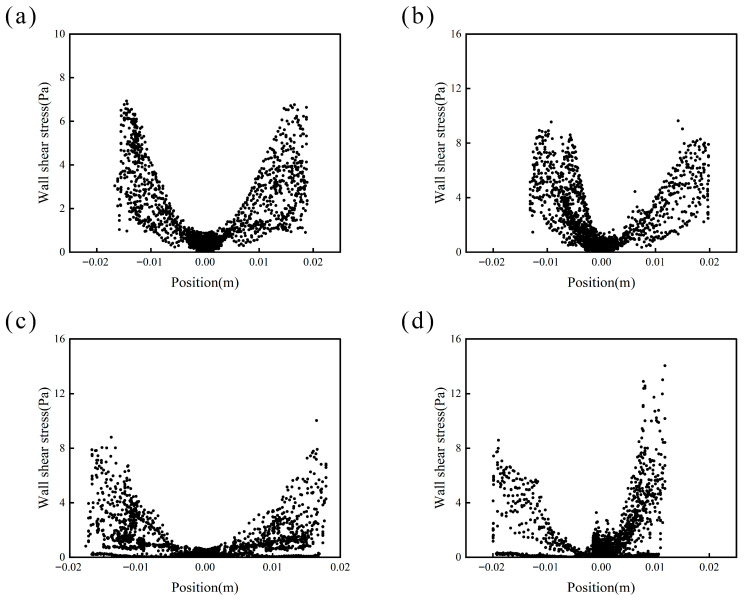
Shear stress field distribution on the impeller plane at different rotational speeds (numerical simulation, captured at t = 20 s): (**a**) 550 rpm, (**b**) 650 rpm, (**c**) 750 rpm, (**d**) 850 rpm.

The simulation results of the blade shear stress field at the four rotational speeds (Figure 12) again reveal significant differences. According to the principle of action and reaction, the blade shear stress indirectly reflects the shear stress distribution in the fluid domain. At 650 rpm, the shear stress is observed to be both uniform and smooth while maintaining adequate intensity. This uniformity and smoothness indicate favorable droplet dispersion within the system, while the magnitude of shear stress indirectly correlates with the size of the microcapsules formed. Shear stress and vortex intensity exhibit mutual influence. When shear stress exceeds a critical threshold, it disrupts the fluid flow within the system, inducing vortex formation that leads to the uneven dispersion of droplets. The distribution of the shear stress field plays a crucial role in determining the particle size distribution of the resulting microcapsules. Excessive shear stress prevents the adequate encapsulation of oil droplets by the wall-forming materials, resulting in incomplete or structurally compromised microcapsules. Insufficient shear stress leads to non-uniform emulsion droplets, making microencapsulation challenging and resulting in a bimodal particle size distribution. To ensure adequate droplet dispersion and achieve uniform microcapsule size during the preparation process, it is essential to maintain both sufficient shear intensity and a homogeneous, smooth shear distribution. Therefore, the optimal rotational speed for obtaining microcapsules with consistent particle size is 650 rpm.

**Figure 13:**
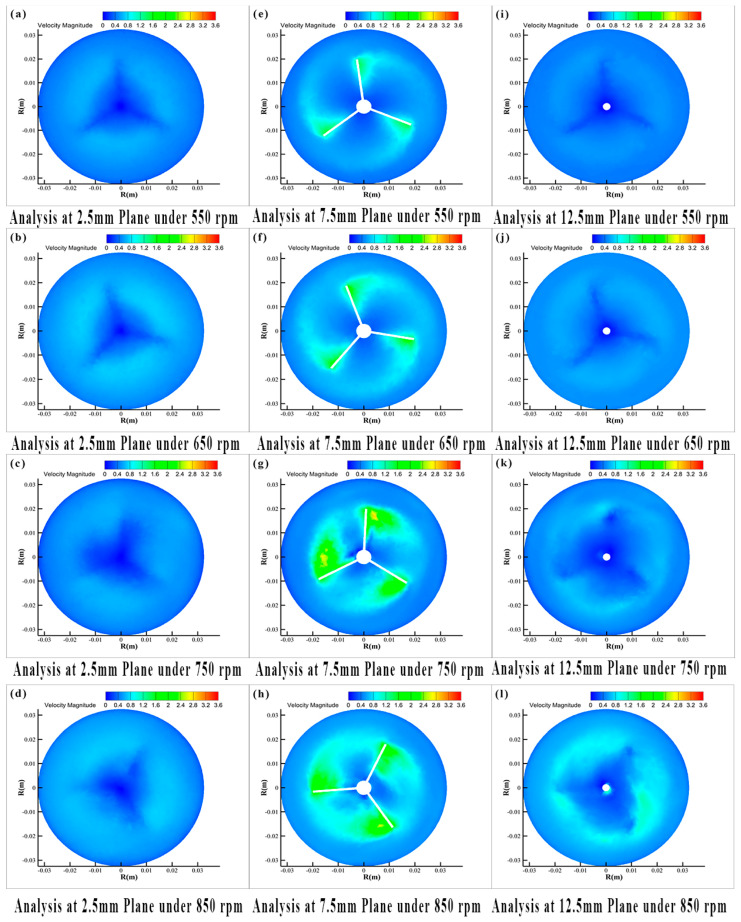
Radial velocity components across horizontal planes at different rotational speeds (numerical simulation, captured at t = 20 s): (**a**–**d**) At 2.5 mm height for 550–850 rpm, (**e**–**h**) at 7.5 mm height for 550–850 rpm, (**i**–**l**) at 12.5 mm height for 550–850 rpm.

**Figure 14:**
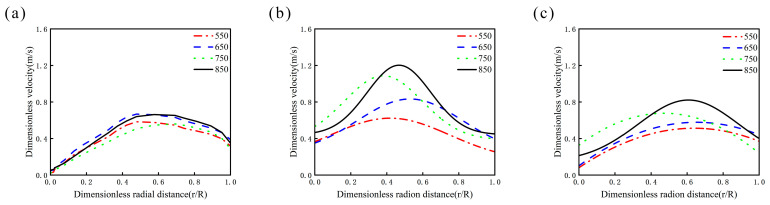
Radial velocity components on the horizontal plane at different rotational speeds (numerical simulation, captured at t = 20 s): (**a**) 2.5 mm above the reactor base; (**b**) 7.5 mm above the reactor base; (**c**) 12.5 mm above the reactor base.

Figure 13 and Figure 14 illustrates the radial velocity components of the model across different horizontal planes and rotational speeds. Divide the radius by the radius (r) of the beaker, and different line segments represent different rotational speeds. At z = 2.5 mm, corresponding to the mid-plane between the beaker bottom and impeller, the radial velocity component gradually increases with radius, reaching its maximum near the impeller tip. In the cross-section at z = 7.5 mm, corresponding to the impeller mid-plane, the maximum radial velocity shifts toward the impeller center. This shift occurs due to the presence of numerous small bubbles near the impeller tip creating low-pressure zones, resulting in uneven fluid distribution and consequently displacing the velocity maximum toward the impeller center. At the z = 12.5 mm plane, the maximum radial velocity shifts back to the vicinity of the impeller tip.

Analysis of the velocity profiles across all three planes clearly shows that the curves at 550 rpm and 650 rpm are nearly identical, indicating relatively uniform fluid velocities in all directions and system stability prior to cavitation onset. When the rotational speed increases to 750 rpm, the profile shape undergoes significant changes, particularly exhibiting a sudden variation at the 7.5 mm plane. This phenomenon occurs because elevated rotational speeds entrain air into the system through impeller action. The introduced air promotes vortex breakdown and causes the self-circulation zone beneath the impeller to shift upward (Figure 7), collectively contributing to the intensified radial velocity component observed at the 7.5 mm plane.

The velocity components significantly influence both the fluid dispersion efficiency and the resultant microcapsule size distribution. At 650 rpm, the system maintains stability while exhibiting relatively high velocity components across all directions. Comparative analysis within the 550–850 rpm range confirms that 650 rpm provides the most uniform velocity distribution, consequently yielding microcapsules with the narrowest particle size distribution.

## 5. Conclusions

The internal flow characteristics of the stirrer are extremely complex, attributed to its high rotational speed, strong turbulence intensity, and other inherent features, which necessitates the consideration of multiple influencing factors. In this study, the Standard k-ε model and the VOF multiphase flow method were employed to perform three-dimensional numerical simulations of the gas–liquid two-phase flow inside the stirrer, with the numerical method validated accordingly. The influence of different rotational speeds on the internal flow field of the stirrer under specific dimensions was investigated to determine the optimal rotational speed. Meanwhile, microcapsules were prepared experimentally under the corresponding emulsification rotational speeds. The results obtained from the numerical simulations are in good agreement and coupling with the experimental findings. The main research contents are summarized as follows:

Through systematic simulation and analysis of the gas–liquid two-phase flow field within the agitated reactor under varying rotational speeds, this study provides a comprehensive hydrodynamic explanation for the impact of agitation intensity on microcapsule uniformity. The principal findings are summarized as follows:

1. The critical speed for vortex breakdown occurs at approximately 750 rpm. The initial vortex rupture emerges at two specific locations: near the vessel center at height y/H = 0.172 and approximately 0.02 m from the center. These positions simultaneously exhibit peak values in both turbulent kinetic energy and vortex dissipation rate. Below 750 rpm, the vorticity within the flow field increases in an organized manner with rising rotational speed. As the speed approaches 750 rpm, the vortices expand to their maximum extent, reaching the critical state for vortex breakdown. Beyond this threshold, further speed elevation triggers extensive vortex rupture, resulting in substantial flow field destabilization. This intensified fluid chaos enhances droplet-impeller collisions, promoting droplet fragmentation and consequently reducing microcapsule uniformity. These numerical predictions show excellent agreement with experimental observations.
2. The combined effects of increased flow velocity and enhanced turbulent kinetic energy induce severe cavitation and irregular turbulence within the reactor. The cavitation phenomenon generates cavitation bubbles, forming microscopic vapor cavities that progressively expand throughout the liquid phase. When subjected to impeller-induced shear forces, these cavities undergo violent collapse, significantly disturbing the flow field and consequently compromising microcapsule uniformity.
3. The onset of cavitation substantially alters the volume distribution of both liquid and gas phases. A noticeable decrease in liquid height occurs alongside transformation of the phase interface from a well-defined funnel shape to an irregular morphology, consistent with experimental observations. Under high-speed impeller rotation, the entrainment of upper gaseous phase into the lower liquid phase creates low-pressure regions within bubbles, causing liquid accumulation near the impeller and subsequent liquid level depression.

When the rotational speed exceeds 650 rpm, the simultaneous occurrence of vortex breakdown and cavitation phenomena causes substantial flow field destabilization, thereby reducing microcapsule uniformity. For operations requiring speeds above 650 rpm while maintaining product uniformity, implementing vortex and cavitation suppression strategies becomes essential. Effective mitigation approaches include installing mesh screens [31] between the impeller and reactor wall, incorporating turbulence rods, or modifying impeller geometry to optimize flow patterns.

## Figures and Tables

**Table 1:** Raw materials and core purposes for microcapsule preparation.

Material Name	Specification/Purity	Manufacturer	Core Experimental Purpose
Epoxy Resin-128	Industrial grade	Guangzhou Yuanyi Chemical Co., Ltd., Guangzhou, China.	Wall material monomer for synthesizing urea-formaldehyde-melamine prepolymer
Urea (U)	Industrial grade	Beijing Kangpuhuiwei Technology Co., Ltd., Beijing, China	Wall material monomer for synthesizing urea-formaldehyde-melamine prepolymer
Melamine (M)	Industrial grade, purity ≥ 99.8%	Xinjiang Yihua Chemi-cal Industry Co., Ltd., Changji, China	Wall material modifying monomer participating in polycondensation of prepolymer
Formaldehyde solution (F)	37% industrial grade	Guangzhou Jinwang Chemical Co., Ltd., Guangzhou, China	Wall material monomer reacting with urea and melamine via polycondensation to form urea-formaldehyde-melamine resin prepolymer
Sodium Chloride	Analytical grade	Xilong Scientific Co., Ltd., Shantou, China	Osmotic pressure regulator for emulsion system, maintaining stability of oil-in-water emulsion
Polyvinyl Alcohol (PVA)	Analytical grade	Shanghai Macklin Biochemical Technology Co., Ltd., Shanghai, China	Non-ionic emulsifier to reduce interfacial tension between oil and water phases, promoting uniform dispersion of epoxy resin in aqueous phase
EMA Polyethylene Maleic Anhydride	Analytical grade	Hubei Baidu Chemical Co., Ltd., Wuhan, China	Polymeric emulsifying dispersant to improve suspension stability of emulsion system and avoid agglomeration and sedimentation of emulsion droplets
Resorcinol	Industrial grade, purity ≥ 99.7%	Zhejiang Hongsheng Chemical Co., Ltd., Shaoxing, China.	Curing crosslinking agent for prepolymer, promoting in situ crosslinking and curing of urea-formaldehyde-melamine resin
3-(Methacryloyloxy)-propyltrimethoxysilane (KH570)	Analytical grade	Shanghai Macklin Biochemical Technology Co., Ltd., Shanghai, China.	Coupling agent to improve interfacial compatibility between epoxy resin core material and resin capsule wall
Sodium Hydroxide (NaOH)	Analytical grade	Sinopharm Chemical Reagent Co., Ltd., Shanghai, China.	pH adjuster to regulate system pH during prepolymer synthesis stage
Dilute Sulfuric Acid	Analytical grade	Sinopharm Chemical Reagent Co., Ltd., Shanghai, China.	pH adjuster to regulate system pH during in situ polymerization stage

**Table 2:** Characteristic Values of Reynolds Number and Weber Number of the Stirring System at Different Rotational Speeds.

Stirring Speed (rpm)	$\mathrm{Rotational}\;\mathrm{Speed}\;\left(r/s\right)$	Reynolds Number Re	Weber Number We	Flow Regime
550	9.17	14,620	76.7	Fully Turbulent
650	10.83	17,280	107.1	Fully Turbulent
750	12.50	19,940	142.6	Fully Turbulent
850	14.17	22,600	183.2	Fully Turbulent

**Table 3:** Dimensions of the stirred tank model used for analysis.

Parameter Symbol	*D*	*d*	*H*	*h*	*h_m_*	*h_a_*
Size (mm)	65	40	90	50	10	5

**Table 4:** Core parameters of mesh schemes and quality.

Element Size (mm)	Node Count	Cell Count	Max Aspect Ratio	Min Orthogonality Quality
1.2	89,245	486,102	5.21	0.282
1.0	156,328	857,246	4.98	0.305
0.8	267,712	1,450,121	4.76	0.321
0.6	498,654	2,763,418	4.23	0.354

**Table 5:** Verification results of grid convergence study (at the optimal rotational speed of 650 rpm).

Element Size (mm)	Max TKE (m^2^/s^2^)	Average Velocity (m/s)	Average Shear Stress (Pa)	Max GCI Value
1.2	0.0215	0.382	1.05	
1.0	0.0243	0.415	1.18	8.27%
0.8	0.0258	0.432	1.25	1.08%
0.6	0.0262	0.436	1.26	0.42%

**Table 6:** Supplementary verification results of grid convergence study (at the maximum rotational speed of 850 rpm).

Element Size (mm)	Max TKE (m^2^/s^2^)	Average Velocity (m/s)	Average Shear Stress (Pa)	Max GCI Value
1.2	0.0312	0.514	1.47	
1.0	0.0348	0.557	1.62	7.85%
0.8	0.0369	0.581	1.71	0.96%
0.6	0.0373	0.585	1.72	0.38%

**Table 7:** Detailed boundary settings of the computational domain.

Boundary Name	Boundary Type	Boundary Category (Dirichlet/Neumann)
Tank bottom	No-slip wall	Dirichlet
Tank sidewall	No-slip wall	Dirichlet
Gas–liquid free surface	Pressure outlet + free surface	Neumann + Dirichlet
Top gas outlet	Pressure outlet	Neumann
Impeller wall	No-slip wall	Dirichlet
Impeller hub wall	No-slip wall	Dirichlet

**Table 8:** Time Step Independence Verification Results.

Verification Scheme	Time Step (s)	Single-Step Rotation Angle	Average Velocity (m/s)	Maximum TKE (m^2^/s^2^)	Stable Liquid Level Height (mm)	Average Relative Deviation of Parameters
1	4.0 × 10^−4^	2°	0.418	0.0249	51.2	3.25%
2	2.0 × 10^−4^	1°	0.432	0.0258	50.6	0
3	1.0 × 10^−4^	0.5°	0.435	0.0261	50.4	0.68%

## Data Availability

The original contributions presented in this study are included in the article. Further inquiries can be directed to the corresponding author.
